# Editorial: Speech-language neurophysiology and intervention: neural markers, predictors, and correlates of treatment response

**DOI:** 10.3389/fnhum.2024.1473370

**Published:** 2024-08-14

**Authors:** Maja Rogić Vidaković, Brooke-Mai Whelan

**Affiliations:** ^1^Laboratory for Human and Experimental Neurophysiology, Department of Neuroscience, School of Medicine, University of Split, Split, Croatia; ^2^School of Health and Rehabilitation Sciences, The University of Queensland, Brisbane, QLD, Australia

**Keywords:** speech, language, neural markers, treatment response, predictors

Acquired communication disorders, resulting from events such as strokes, traumatic brain injuries, or progressive neurological conditions, can severely impair a person's ability to speak, understand, read, and write. These disorders can significantly impact the ability to engage in everyday interactions, leading to challenges in personal relationships, educational attainment, and professional success. Effective rehabilitation strategies are essential not only for restoring communication abilities but also for improving overall quality of life. Furthermore, mitigating adverse effects on speech and language during neurosurgical procedures, such as brain tumor resection, is a critical aspect of patient care.

The optimal mapping of speech and language functional brain cortices during awake craniotomy and determining behavioral treatment responses to speech-language therapy in patients with acquired brain injuries necessitates a deep understanding of the underlying neurophysiological mechanisms of speech and language processing. Examining brain activity patterns and connectivity before, during, and/or after therapy, may lead to the identification of neural biomarkers that can predict treatment outcomes and inform personalized approaches to intervention. Also, damage to critical speech and language brain areas and white matter tracts can be prevented by functional brain mapping procedures during neurosurgery.

This Research Topic showcases three original research publications and one brief research report that explore behavioral and neurophysiological substrates of communication disorders in patients receiving behavioral interventions following acquired brain injuries, as well as predictor variables for awake craniotomy. This body of research provides insights into optimal patient selection for awake craniotomies, the feasibility of telepriming sentence production in aphasia, the application of visual P300 brain computer interface spelling to aphasia rehabilitation, and the influence of speech treatment for hypokinetic dysarthria on language production.

Kram et al. identified specific predictor variables for the selection of patients undergoing awake craniotomy, in relation to pre-operative language status, patient, and tumor characteristics. Higher preoperative language eloquence levels, lower age, and higher number of paraphasias were predictors for awake surgery. These findings indicate the need for future studies to systematically compare preoperative and intraoperative speech and language performance, to verify the impact of neurosurgical procedures on language status and possible predictors of post-operative speech and language status. Thus far, no standardized, objective system has been developed for selecting patients (taking into account specific characteristics of transparent and non-transparent languages) undergoing awake craniotomies with mapping of speech and language eloquent brain cortices.

Behavioral interventions are crucial for rehabilitating speech and language disorders following acquired brain injury. These interventions, which include therapies designed to improve speech production, language comprehension, and communication strategies, play a vital role in helping individuals regain their communicative abilities. Recent advancements in methodologies for evaluating treatment responsiveness have significantly enhanced our ability to tailor and optimize these interventions. Neurophysiological assessment techniques such as event-related potentials (ERPs) provide valuable insights into the neural underpinnings of speech and language processing. By measuring brain responses to specific stimuli, ERPs allow clinicians to objectively assess the effectiveness of behavioral therapies and make data-driven adjustments to treatment plans. This integration of advanced neurophysiological tools to evaluate the effects of behavioral interventions promises to improve outcomes for patients, enabling more precise and personalized approaches to speech and language rehabilitation.

A further intervention study by Kleih and Botrel examined the utility of a P300 brain computer interface (BCI)-based spelling program, to improve attention, language production, psychosocial wellbeing and quality of life in individuals with motor aphasia following stroke. The results of this study were largely inconclusive due to a small sample size and absence of a control group, despite the observation of significant improvements in spontaneous speech and quality of life following BCI-based intervention. The need for further research in this area to explore the potential utility of P300 BCI-based programs in aphasia rehabilitation was emphasized.

Recently, Lee et al. demonstrated the feasibility of a telepriming application to sentence production rehabilitation in aphasia. Robust priming effects were observed during structural priming tasks delivered via videoconferencing to people with mild to moderate aphasia following left hemisphere stroke. These findings indicate telehealth applications may provide a viable, service delivery model for aphasia interventions, which is especially important for individuals that may not be able to attend in-person treatment sessions.

Finally, Ramage et al. investigated whether a motor learning-based speech treatment [i.e., Lee Silverman Voice Treatment (LSVT LOUD®)] for impaired vocal function in Parkinson's disease (PD) could improve language skills. Despite evidence of language alterations in PD, specifically diminished verb processing, the current study's results did not support the hypothesis that interventions targeting motor speech subsystems would result in improved verb access in individuals with PD. These findings highlight the need for future research to explore alternative approaches for addressing language deficits in PD.

In summary, the collection of studies discussed in this Research Topic, underscores the complexity of effective rehabilitation strategies for acquired communication disorders. The research emphasizes the importance of collaborative efforts between neurosurgeons and speech-language pathologists to mitigate adverse effects on speech and language during neurosurgical procedures, particularly through advanced intraoperative monitoring techniques. The investigation into various interventions, including the Lee Silverman Voice Treatment (LSVT LOUD®) for Parkinson's disease and telehealth applications for aphasia, highlights both the potential and limitations of these methods. While some interventions showed promise, such as telepriming for sentence production in aphasia, others, like the motor learning-based speech treatment for Parkinson's disease, did not yield the expected improvements in language skills. Additionally, studies exploring the use of P300 brain-computer interface spelling programs for aphasia rehabilitation indicate a need for further research due to inconclusive results. This body of work provides valuable insights into patient selection for awake craniotomies and the application of neurophysiological assessment techniques, advocating for more personalized and data-driven approaches to speech and language rehabilitation.

